# Predictive significance of surgery-induced lymphopenia on the survival after curative resection for locally advanced gastric cancer: a retrospective cohort analysis

**DOI:** 10.1186/s12957-023-02887-0

**Published:** 2023-01-16

**Authors:** Masaki Aizawa, Hiroshi Yabusaki, Atsushi Matsuki, Takeo Bamba, Satoru Nakagawa

**Affiliations:** Department of Digestive surgery, Niigata Cancer Center Hospital, 2-15-3, Kawagishicho, Niigata City, Niigata 951-8566 Japan

**Keywords:** Gastric cancer, Prognosis, Postoperative inflammatory response, Lymphocyte

## Abstract

**Background:**

Following the establishment of the anti-cancer effect of immune checkpoint inhibitors, lymphopenia has attracted attention as a parameter of preexisting cancer-related immune tolerance. Although the pretreatment absolute lymphocyte count (ALC) has been reported as a prognostic factor in gastric cancer patients, the impact of perioperative changes in the ALC remains unknown. The aim of the present study was to explore the relationship between surgery-induced lymphopenia and outcome.

**Methods:**

Database entries for 584 patients who underwent curative resections for pathological Stage IB-III gastric cancer were reviewed. We retrospectively compared clinicopathological factors including pretreatment ALC (pre-ALC) and ALC at first visit after discharge (post-ALC) with the survival. The low ALC was defined as < 1000/μL.

**Results:**

The ALC decreased significantly at 1 and 3 days after surgery and then recovered to the baseline value. A low pre-ALC (*p* < 0.001) and a low post-ALC (*p* < 0.001) were both correlated with a poor relapse-free survival (RFS). A multivariate analysis of RFS identified a low post-ALC (hazard ratio 1.875, 95% CI 1.156–3.402, *p* = 0.01), age, gender, BMI, T disease, N disease, severe vessel invasion, type of gastrectomy and postoperative morbidity as independent factors. The low post-ALC group had a poor RFS among patients with Stage II (*p* = 0.04) and Stage III (*p* = 0.04) disease, but not among patients with Stage IB disease (*p* = 0.13). Consistently, the overall survival (OS) rate was significantly lower among patients with a low post-ALC for all stage (*p* < 0.001), stage II (*p* = 0.02) and stage III (*p* = 0.01) disease, not for stage IB (*p* = 0.09). A low post-ALC was identified as an independent factor for predicting OS by multivariate analysis (hazard ratio: 2.275, 95% CI 1.373–3.769, *p* = 0.01).

**Conclusions:**

A decrease in post-ALC was correlated with both of RFS and OS after curative resection in patients with locally advanced gastric cancer.

**Highlights:**

Postoperative lymphopenia was a poor prognostic factor for gastric cancer.

## Background

Gastric cancer is common as a cause of cancer-related death worldwide [1]. Though the curative resection is the most promising treatment for a cure, patients with locally advanced gastric cancer often die of recurrence after surgery [2]. The pathological tumor-node-metastasis (TNM) stage is reliable indicator of the possibility of residual foci of cancer during potentially curative resection, from which the recurrence is supposed to arise. Adjuvant chemotherapy is established under the concept to treat remnant lesions and prevent the recurrence, then randomized controlled trials (RCTs) clearly showed the efficacy [3, 4]. However, the process by which residual micro-metastases develop into recurrences requires clarification for the further improvement of multimodal treatments.

The host inflammatory response is thought to play an important role in cancer development and progression, and host immunocytes are an essential component of the tumor microenvironment [5, 6]. Lymphopenia is considered a parameter of preexisting cancer-related immune tolerance. Several studies have reported that the pretreatment absolute lymphocyte count (ALC), which can be estimated by performing a peripheral blood examination at baseline, was significantly correlated with the prognosis of patients with solid cancers [7–10].

Surgical trauma is known to induce an inflammatory cascade composed of systemic inflammatory response syndrome (SIRS) and a subsequent anti-inflammatory response known as compensatory anti-inflammatory response syndrome (CARS) [11–13]. The ALC is known to decrease temporarily after surgery, reflecting the degree of CARS [14]. This series of responses might influence the development of recurrences. Practically, the postoperative complication accompanying with excessive inflammatory response after gastrectomy for gastric cancer reported to impair survival [15, 16]. On the other hand, the relationship between postoperative CARS and the recurrence has been unknown.

Recently, the potent efficacy of immune checkpoint inhibitors (ICI) for the treatment of advanced gastric cancer, with the aim of regulating immune tolerance, has been established [17–19]. The ALC [20–22] and the neutrophil-to-lymphocyte ratio (NLR) [23–25] have been the focus of attention as prognostic biomarkers for ICI treatment.

The aim of the present study was to investigate the impact of the perioperative ALC on the outcomes of patients who underwent curative resections for locally advanced gastric cancer.

## Methods

### Patients

This study was conducted as a retrospective analysis of clinical data from a prospectively maintained database of Niigata Cancer Center Hospital. Patients with pathologically diagnosed Stage IB-III gastric cancer who underwent gastrectomy with curative intent between January 2006 and December 2019 were enrolled. The exclusion criteria were as follows: (1) use of preoperative chemotherapy, (2) remnant gastric cancer, (3) any evidence of residual tumor, (4) simultaneous active malignancy in another organ, (5) simultaneous surgery for other disease, (6) postoperative hospital death, and 7) unavailability of blood examination data collected during a period corresponding to postoperative day (POD) 15–60.

### Data collection

Data on clinical variables including age, sex, BMI, tumor location, representative histological feature, surgical findings, postoperative morbidity, pathological findings, pathological TNM stage, presence or absence of postoperative chemotherapy, and compliance with postoperative chemotherapy were collected. The TNM stage was defined according to the Japanese classification of gastric carcinoma, 3rd English edition [26], and the Union for International Cancer Control TNM classification of malignant tumors, 8th edition [27]. ALC was appraised at baseline and on POD 1, POD 3, and POD 7 as well as at the time of the first clinical visit after hospital discharge. In cases with hospitalization for 30 days or more, data obtained at around POD 30 was substituted for that of the first visit date. The median (range) duration from surgery until the day of the first clinical visit or the substituted examination date was 31 (17–60) days. After discharge, patients visited the outpatient clinic every 1–3 months for the first 2 years and every 3–6 months thereafter. The date on which the first recurrence after surgery was diagnosed and the site of the recurrence as determined using relevant imaging was retrieved from the medical records.

### Statistical analysis

All continuous variables were presented as medians and ranges. The ALC was compared in relation to the category and postoperative period using the Mann–Whitney *U* test and the Wilcoxon’s test, respectively. Relapse-free survival (RFS) was defined as the number of months from surgery until relapse or death from any cause. Overall survival (OS) was defined as the number of months from surgery until death from any cause. RFS and OS were assessed using a Kaplan–Meier analysis, respectively. The log-rank test was used for comparisons of survival between two groups. Variables that were significantly correlated with the survival in a univariate analysis were further applied in a multivariable Cox model and subgroup analyses. A *p* value < 0.05 was considered to denote statistical significance. The statistical analyses were performed using a statistical analysis software package (SPSS 9.0, SPSS, Inc., Chicago, IL).

### Declarations

All procedures were in accordance with the ethical standards of the responsible committees on human experimentation (institutional and national) and with the Helsinki Declaration of 1964 and later versions. The study was approved by the institutional review board of Niigata Cancer Center Hospital (2020–231). Informed consent was obtained from all individual participants in the form of opt-out.

## Results

### Clinicopathological characteristics

A total of 584 patients were enrolled in this study. The baseline characteristics, tumor-related factors, and perioperative findings are shown in Table 1. The median age (range) was 67 (21–92) years, and the study population was predominantly male (67.5%). The pathological stage was diagnosed as pStage IB in 163 (27.9%) patients, pStage II in 236 (40.4%) patients, and pStage III in 185 (31.6%) patients. Postoperative adjuvant chemotherapy was administered in 361 (61.8%) patients. Ninety-four (16.1%) patients developed recurrences during the observation period. The median follow-up period was 59.2 months.

**Table 1:** Patients’ characteristics, tumor related factor and surgical factors

Factors		Number	(%)
Age (years)	Median [range]	67	[21–92]
Sex	Male Female	394 190	(67.5) (32.5)
Body mass index	Median [range]	23.0	[14.0–36.7]
Tumor location	Upper 1/3 stomach Middle 1/3 stomach Lower 1/3 stomach Whole stomach	142 240 198 4	(24.3) (41.1) (33.9) (0.7)
Histological features	Differentiated Un-differentiated	330 254	(56.5) (43.5)
Pathological T disease	T1 T2 T3 T4	83 184 160 157	(14.2) (31.5) (27.4) (26.9)
Pathological N disease	N0 N1 N2 N3	204 194 99 87	(34.9) (33.2) (17.0) (14.9)
Pathological stage	IB IIA IIB IIIA IIIB IIIC	163 102 134 82 56 47	(27.9) (17.5) (22.9) (14.0) (9.6) (8.0)
Surgical approach	Laparoscopic Open method	104 480	(17.8) (82.2)
Surgical method	Distal gastrectomy Total gastrectomy Proximal gastrectomy Segmental gastrectomy	401 167 13 3	(68.7) (28.6) (2.2) (0.5)
Lymph node dissection	D1 + D2 D2 +	182 371 31	(31.2) (63.5) (5.3)
Resection of adjacent organ	Spleen Pancreas Lower thoracic esophagus Transverse colon Liver Uterus, ovary	27 9 12 1 1 4	(4.6) (1.5) (2.1) (0.2) (0.2) (0.7)
Operation time (min)	Median [range]	165	[65–678)]
Bleeding (mL)	Median [range]	70	[2,690]
Postoperative morbidity (Clavien-Dindo classification)	None Grade I Grade II Grade IIIa Grade IIIb Grade IVa Grade IVb	493 8 28 46 5 2 2	(84.4) (1.4) (4.8) (7.9) (0.9) (0.3) (0.3)
Postoperative adjuvant chemotherapy	Present Absent	362 222	(62.0) (38.0)

### Perioperative ALC values

**Fig. 1:**
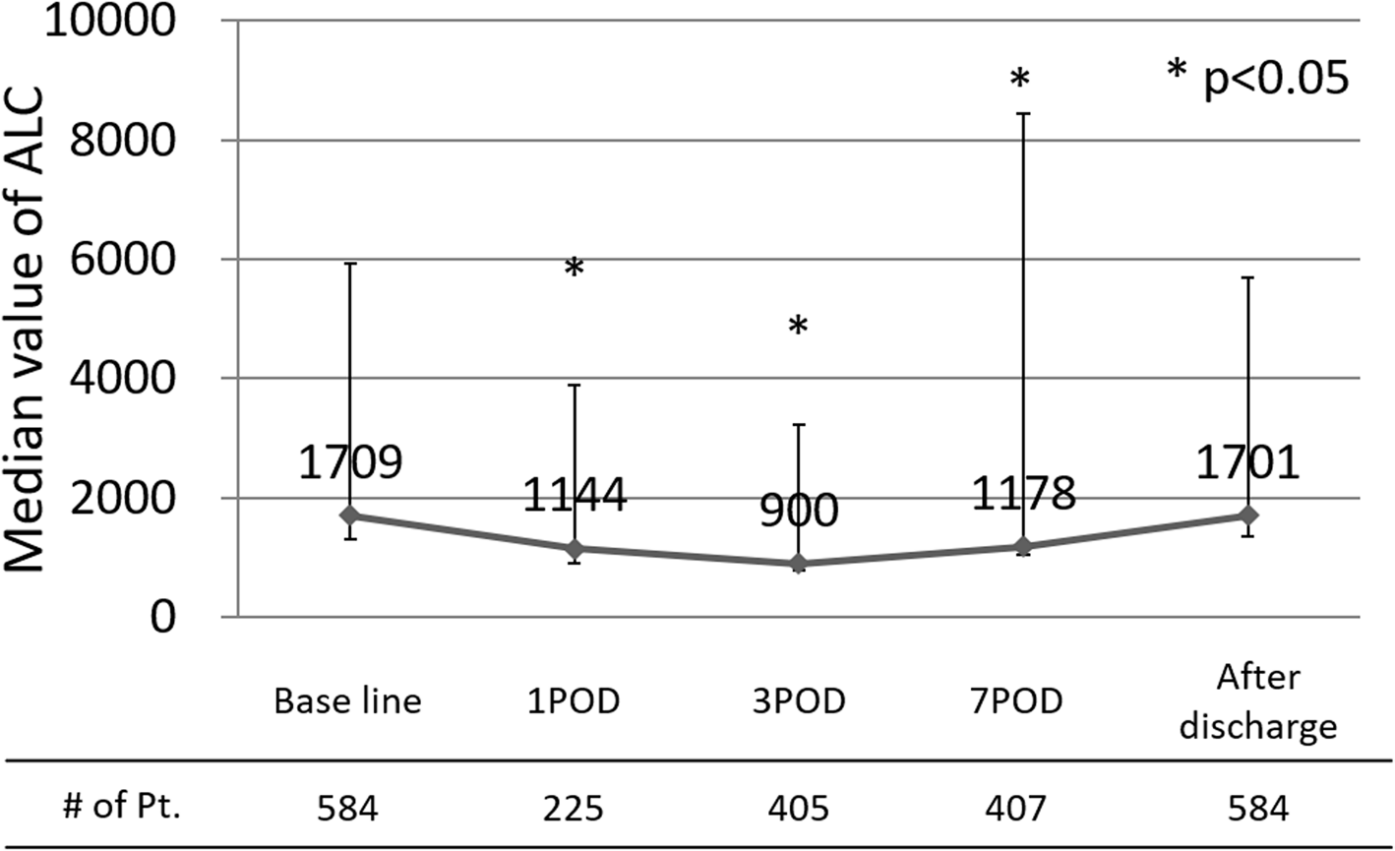
ALC values during the perioperative course. The values were compared using the Wilcoxon’s test

The perioperative change in ALC is shown in Fig. 1. The postoperative ALCs were significantly lower than the baseline ALC (pre-ALC). The decline in ALC bottomed out on POD 3 and then began to recover, returning to near baseline. We used representative ALC data obtained after discharge to evaluate the impact of the postoperative ALC (post-ALC) on the survival outcome. The pre- and post-ALC values assessed for patients in each clinicopathological variable category are shown in Table 2. While the pre-ALC values were correlated with body mass index (BMI) and vessel invasion, the representative post-ALC values were correlated with BMI alone.

**Table 2:** Clinicopathological variables and perioperative ALC values

Factors		Number	Pre-ALC median [range]	*p* value	Post-ALC median [range]	*p* value
Age (years)	< 70 ≥ 70	347 237	1747 [588–4229] 1732 [591–4001]	0.07	1732 [592–4001] 1663 [357–3559]	0.06
Sex	Male Female	394 190	1669 [588–3531] 1730 [396–4229]	0.46	1676 [637–3557] 1736 [357–4001]	0.19
Body mass index	< 25.0 ≥ 25.0	414 170	1854 [655–3952] 1681 [396–4226]	< 0.01	1783 [616–4001] 1647 [357–3559]	< 0.01
Tumor size	< 8 cm ≥ 8 cm	504 80	1713 [396–4229] 1656 [588–3192]	0.24	1712 [357–4001] 1644 [363–3332]	0.16
Depth of tumor	pT1–2 pT3–4	267 317	1710 [630–4229] 1707 [396–3729]	0.34	1724 [357–4001] 1700 [363–3507]	0.60
Nodal status	pN0–1 pN2–3	398 186	1734 [396–4229] 1682 [655–3492]	0.13	1732 [357–4001] 1645 [363–3461]	0.11
Vessel invasion	Ly0–2 and V0–2 Ly3 and/or V3	481 101	1742 [396–4229] 1587 [655–3952]	< 0.01	1729 [357–4001] 1627 [531–2790]	0.14
Histological feature	Differentiated Un-differentiated	330 254	1724 [588–4229] 1695 [396–3952]	0.36	1721 [357–4001] 1689 [363–3557]	0.51
Type of gastrectomy	Non-total Total	417 167	1702 [588–4229] 1735 [396–3952]	0.55	1724 [357–4001] 1652 [531–3557]	0.29
Surgical approach	Open method Laparoscopic	480 104	1690 [396–4229] 1755 [616–3729]	0.35	1687 [357–4001] 1805 [630–3461]	0.25
Operation time	≤ 240 min > 240 min	449 135	1686 [396–4229] 1792 [600–3952]	0.43	1693 [357–4001] 1739 [624–3559]	0.58
Blood loss	≤ 200 mL > 200 mL	477 107	1700 [396–4229] 1808 [588–3952]	0.91	1700 [357–4001] 1705 [624–3493]	0.92
Resection of other organs	( −) ( +)	537 47	1710 [396–4229] 1659 [588–3072]	0.51	1701 [357–4001] 1652 [658–3494]	0.88
Morbidity CD grade	< Grade II ≥ Grade II	498 86	1695 [396–4229] 1817 [663–3952]	0.17	1701 [357–3559] 1703 [363–4001]	0.89

A low ALC was defined as < 1000/μL in accordance with the findings of previous reports [28]. While 42 (7.2%) patients were categorized as having a low pre-ALC, 54 (9.2%) patients were categorized as having a low post-ALC.

### Survival analysis

**Fig. 2:**
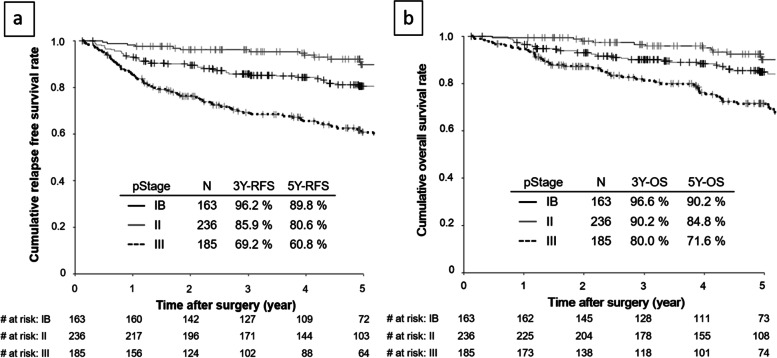
Relapse-free survival after surgery according to pathological stage (**a**). Overall survival after surgery according to pathological stage (**b**)

The RFS and OS curves stratified according to pathological stage are shown in Fig. 2. The 3-year RFS rates in patients with pStage IB, II and III were 96.2%, 85.9%, and 69.2%, respectively. The 5-year OS rates in patients with pStage IB, II and III were 90.2%, 84.8%, and 71.6%, respectively. The frequency of postoperative chemotherapy in the low post-ALC group was significantly lower (25/54; 46.3%) than that in the normal post-ALC group (337/530; 63.6%) (*p* = 0.01). The median time from surgery until the start of chemotherapy was similar: 38 days in the regular post-ALC group, and 35 days in the low post-ALC group. The treatment completion rate in the low post-ALC group (21/25; 84.0%) was higher than that in the normal post-ALC group (266/337; 78.9%; *p* = 0.02).

**Fig. 3:**
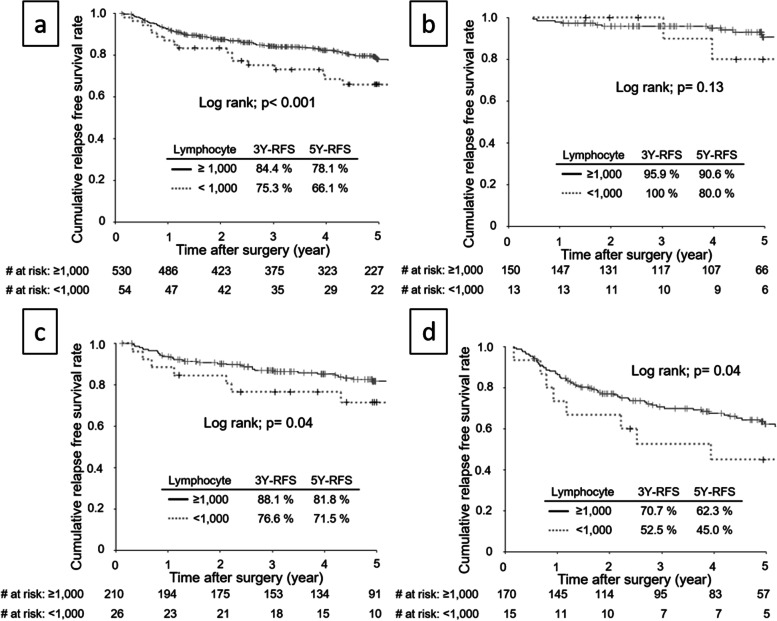
Relapse-free survival curves stratified according to post-ALC values among patients with all stage (**a**), pStage IB (**b**), pStage II (**c**), and pStage III (**d**)

The RFS rate was significantly lower among patients with a low post-ALC for all stage (Fig. 3a), stage II (Fig. 3c), and stage III (Fig. 3d) disease, but not for patients with stage IB disease (Fig. 3b). The results of the univariate and multivariate analyses of RFS are shown in Table 3. Several covariates including age, sex, BMI, tumor size, pT disease, pN disease, vessel invasion, type of gastrectomy, blood loss on surgery, postoperative morbidity (≥ Grade II), low pre-ALC and low post-ALC were significantly correlated with RFS. Among these parameters, age, sex, BMI, tumor size, T disease, N disease, vessel invasion, total gastrectomy, postoperative morbidity (≥ Grade II), and low post-ALC were identified as independent factors predicting relapse.

**Table 3:** Predictive value of covariates for relapse

Covariates		Number	Univariate analysis		Multivariate analysis		
			3Y-RFS (%)	Log rank *P* value	HR	[95% CI]	*p* value
Age (years)	< 70 ≥ 70	347 237	86.5 79.1	< 0.01	1.906	[1.339–2.715]	< 0.01
Sex	Male Female	394 190	80.7 89.5	< 0.01	2.047	[1.327–3.158]	< 0.01
Body mass index	< 25.0 ≥ 25.0	414 170	81.0 89.7	0.04	1.768	[1.165–2.681]	< 0.01
Tumor size	< 8 cm ≥ 8 cm	504 80	86.0 67.3	< 0.01	1.609	[1.061–2.440]	0.03
Depth of tumor	pT1-2 pT3-4	267 317	93.0 75.4	< 0.01	1.711	[1.234–2.742]	< 0.01
Nodal status	pN0-1 pN2-3	398 186	89.9 69.8	< 0.01	1.903	[1.324–2.734]	< 0.01
Vessel invasion	Ly0–2 and V0–2 Ly3 and/or V3	481 101	88.3 60.3	< 0.01	2.257	[1.535–3.317]	< 0.01
Histological feature	Differentiated Un-differentiated	330 254	85.1 81.6	0.30	–	–	–
Type of gastrectomy	Non-total Total	417 167	88.3 71.6	< 0.01	1.598	[1.116–2.290]	0.01
Surgical approach	Open method Laparoscopic	480 104	83.1 87.3	0.31	–	–	–
Operation time	≤ 240 min > 240 min	449 135	83.7 83.6	0.40	–	–	–
Blood loss	≤ 200 mL > 200 mL	477 107	84.7 78.1	0.03	1.029	[0.679–1.561]	0.89
Resection of other organs	( −) ( +)	537 47	84.2 75.9	0.10	–	–	–
Morbidity	< Grade II ≥ Grade II	498 86	85.0 75.0	< 0.01	1.589	[1.060–2.382]	0.03
Pre-ALC	≥ 1000/μL < 1000/μL	524 60	85.0 71.8	< 0.01	0.951	[0.533–1.694]	0.86
Post-ALC	≥ 1000/μL < 1000/μL	521 63	85.9 63.7	< 0.01	1.875	[1.156–3.042]	0.01

**Fig. 4:**
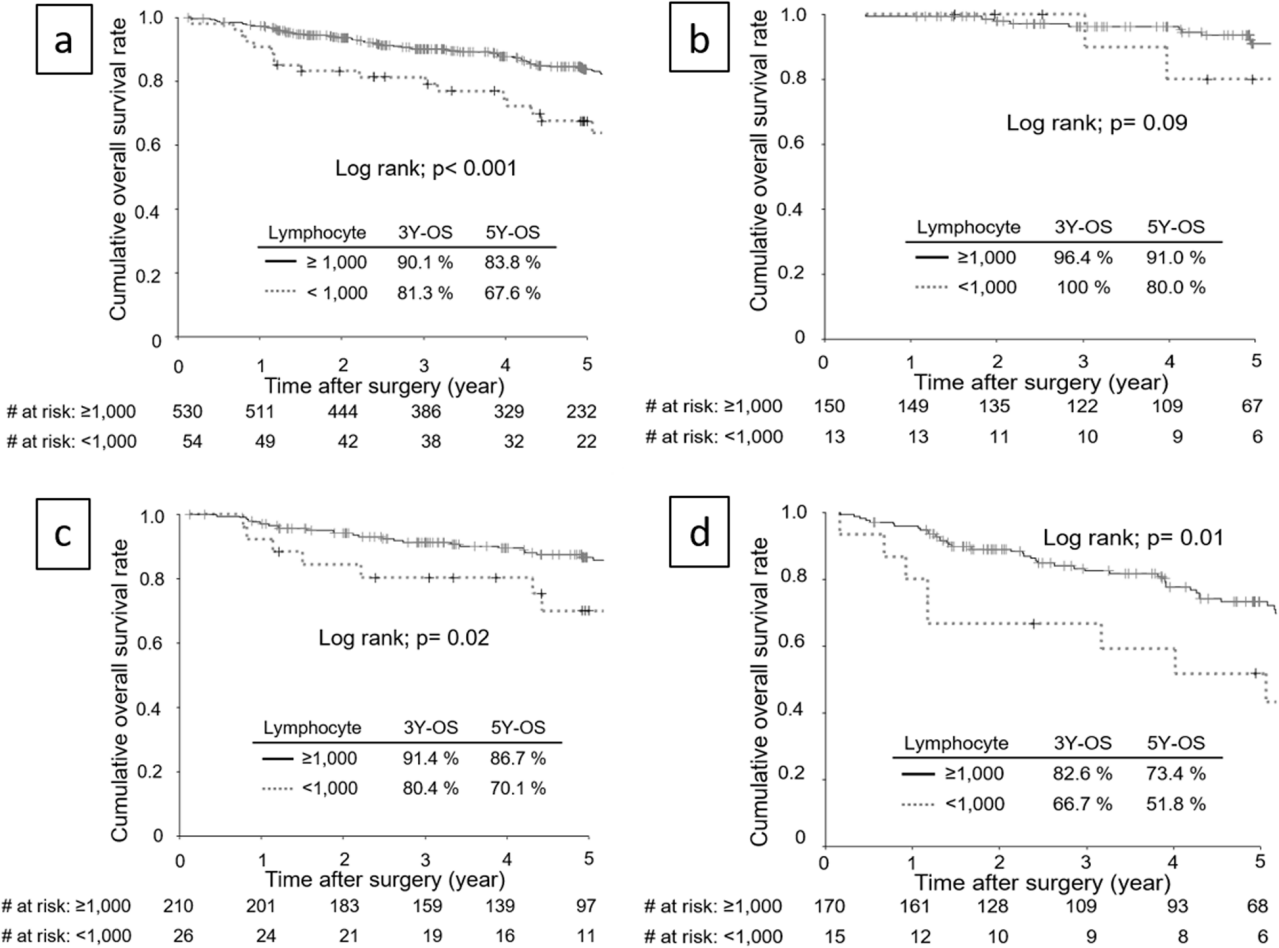
Overall survival curves stratified according to post-ALC values among patients with all stage (**a**), pStage IB (**b**), pStage II (**c**), and pStage III (**d**)

Consistent with RFS, the OS rate was significantly lower among patients with a low post-ALC for all stage (Fig. 4a), Stage II (Fig. 4c) and Stage III (Fig. 4d) disease, not for Stage IB (Fig. 4b). The results of the univariate and multivariate analyses of RFS are shown in Table 4. The age, sex, tumor size, pT disease, pN disease, vessel invasion, type of gastrectomy, postoperative morbidity (≥ Grade II), low pre-ALC and low post- ALC were significantly correlated with OS, then age, sex, tumor size, T disease, N disease, vessel invasion, total gastrectomy, postoperative morbidity (≥ Grade II), and low post-ALC were identified as independent factors.

**Table 4:** Predictive value of covariates for overall survival

Covariates		Number	Univariate analysis		Multivariate analysis		
			5Y-OS (%)	Log rank *P* value	HR	[95% CI]	*p* value
Age (years)	< 70 ≥ 70	347 237	86.4 75.8	< 0.01	2.277	[1.558–3.328]	< 0.01
Sex	Male Female	394 190	79.1 88.8	< 0.01	1.816	[1.143–2.883]	0.01
Body mass index	< 25.0 ≥ 25.0	414 170	87.1 80.2	0.07	–	–	–
Tumor size	< 8 cm ≥ 8 cm	504 80	84.2 68.3	< 0.01	1.587	[1.003–2.512]	0.04
Depth of tumor	pT1-2 pT3-4	267 317	88.1 77.1	< 0.01	1.530	[1.003–2.335	0.04
Nodal status	pN0-1 pN2-3	398 186	86.7 72.5	< 0.01	1.577	[1.059–2.349]	0.03
Vessel invasion	Ly0–2 and V0–2 Ly3 and/or V3	481 101	87.6 76.7	< 0.01	2.444	[1.610–3.709]	< 0.01
Histological feature	Differentiated Un-differentiated	330 254	82.5 81.9	0.65	–	–	–
Type of gastrectomy	Non-total Total	417 167	86.8 70.5	< 0.01	1.518	[1.027–2.244]	0.04
Surgical approach	Open method Laparoscopic	480 104	81.6 90.0	0.28	–	–	–
Operation time	≤ 240 min > 240 min	449 135	81.9 81.5	0.79	–	–	–
Blood loss	≤ 200 mL > 200 mL	477 107	83.9 75.8	0.09	–	–	–
Resection of other organs	( −) ( +)	537 47	83.2 71.5	0.40	–	–	–
Morbidity	< Grade II ≥ Grade II	498 86	83.9 72.5	< 0.01	1.666	[1.088–2.552]	0.02
Pre-ALC	≥ 1000/μL < 1000/μL	524 60	83.4 67.7	0.02	0.900	[0.479–1.688]	0.74
Post-ALC	≥ 1000/μL < 1000/μL	521 63	83.8 67.6	< 0.01	2.275	[1.373–3.769]	0.01

## Discussion

Surgically induced inflammation has been shown to serve as a trigger for the development of distant metastasis, the outgrowth of which had been successfully suppressed preoperatively [29, 30]. The immune escape prompted by the postoperative downregulation of the adaptive immune response is one plausible explanation for this phenomenon. Since lymphocytes play a pivotal role in eradicating cancer cells through the immunological reaction of the host against cancer [31], postoperative lymphopenia is thought to be related to the immune suppressive response of the host, which can encourage the development of recurrence. In the present study, we investigated the effect of postoperative immunosuppression, known as CARS, on the outcomes of patients with Stage IB-III gastric cancer who were suspected of having residual micro-metastases of cancer after surgery.

An assessment of perioperative changes in the ALC (Fig. 1) showed a reduction in ALC values between POD 1 and POD 7, after which the value gradually recovered to the baseline value. Mokart, et al. demonstrated the presence of CARS during the early postoperative period by measuring cytokine levels after surgery in patients with cancer [12]. Rubinkiewicz, et al. reported that the lymphopenia at POD2 after surgery for colorectal cancer occurred in parallel with the decrease of CD4 + lymphocyte, CD8 + lymphocyte and Th17 lymphocyte [32]. Zheng et al. assessed the alteration of lymphocyte subpopulations at POD 3 after gastrectomy for gastric cancer, and an increase in regulatory T cells and the plasma level of TGF-β1, in addition to a decrease in Th17 lymphocytes and a plasma level of IL-17, was observed [33]. A postoperative transient decrease in ALC, which reflected the magnitude of postoperative SIRS and CARS, was consistent with these previous reports.

We focused on the post-ALC measured on around POD 30. It has been reported that sepsis-induced immunosuppressive dysregulation persisted for 28 days [34], and the decrease in this value was considered to be due to the delayed recovery of CARS. The results of the survival analysis showed that the post-ALC was a statistically significant predictor of recurrence that was independent of other known predictive factors and that was more reliable than the pre-ALC. When survival was examined according to each pathological stage, a low post-ALC was significantly correlated with a poor outcome among patients with stage II and III disease, but not among patients with Stage IB disease; this result can probably be attributed to an insufficient number of relapse or death events. Several reports have suggested that the postoperative ALC is related to the long-term outcomes of patients with gastric cancer [35, 36], and the designs of previous studies are not suitable for evaluating patients with remnant cancer or postoperative immunosuppression. Furthermore, survival analyses that include quite a few patients with Stage IA disease have relatively low recurrence rates [35], and the ALC at months after surgery is thought to reflect post-surgery nutrition, rather than the surgery-related immune status [36]. The results of the present study suggested that a 1-month postoperative reduction in ALC was a promising parameter reflecting the dysfunction of the lymphocyte-mediated immune response, which is correlated with the immune tolerance to residual cancer.

The negative effect of surgical morbidity on the survival of gastric cancer patients [15, 16] is also thought to be influenced by the immune status of the patient. The present study identified postoperative morbidity (≥ Grade 2) as another independent factor predicting both the RFS and OS. An excessive elevation of the serum CRP value [37] and a prolonged inflammatory response [38] after a gastrectomy were reportedly associated with a poor prognosis. These results suggest that inflammatory cytokines released by overstimulation of systemic inflammation activated the growth of residual cancerous lesions. However, high-magnitude SIRS enhances subsequent CARS, so postoperative recurrence might develop in response to CARS as well as SIRS.

Following the establishment of the clinical efficacy of ICIs for the treatment of advanced gastric cancer, the additive use of ICIs in perioperative chemotherapy is now being tested [39]. Several reports of treatment with ipilimumab in patients with melanoma have revealed that an increase in the ALC after treatment was correlated with an improved survival outcome [20, 22]. Thus, surgery-induced lymphopenia has the potential to become a treatment target, and recovery of ALC with perioperative treatment may improve survival.

The present study had several limitations. First, as the study was designed retrospectively and was performed at a single institution, the certainty of the evidence remains inadequate. Second, post-ALC was speculated to be an indirect parameter of the immunosuppressive status of the patients, but supportive data was not available. The measurement of lymphocyte subpopulations or the levels of cytokines that act as immunosuppressants in the tumor microenvironment is required. Third, the optimal cut-off value for ALC and the optimal period from surgery until the measurement of immunosuppressive parameters also needs to be elucidated. Fourth, the observational period used to assess long-term survival was insufficient for some of the patients. Fifth, because patients receiving preoperative chemotherapy were excluded from the present study, the value of the post-ALC parameter in this setting remains unclear.

## Conclusions

A decrease in the post-ALC was correlated with both of the RFS and OS after curative resection in patients with locally advanced gastric cancer, regardless of other clinicopathological factors. Low post-ALC may help complement TNM stage in determining adjuvant chemotherapy indications and regimens to further improve the prognosis of stage II and III gastric cancer patients. The future development of treatments focused on postoperative lymphopenia may improve the outcomes of multimodal therapy.

## Data Availability

The datasets generated and analyzed during the current study are not publicly available due to the institutional privacy policy on clinical data but are available from the corresponding author on reasonable request.

## Abbreviations

TNM: Tumor-node-metastasis
RCT: Randomized controlled trial
ALC: Absolute lymphocyte count
pre-ALC: Preoperative ALC
Post-ALC: Postoperative ALC
SIRS: Systemic inflammatory response syndrome
CARS: Compensatory anti-inflammatory response syndrome
ICI: Immune checkpoint inhibitors
NLR: Neutrophil-to-lymphocyte ratio
POD: Postoperative day
TNM: Tumor-node-metastasis
RFS: Relapse-free survival
OS: Overall survival
BMI: Body mass index
